# Gene duplication in an African cichlid adaptive radiation

**DOI:** 10.1186/1471-2164-15-161

**Published:** 2014-02-26

**Authors:** Heather E Machado, Ginger Jui, Domino A Joyce, Christian RL Reilly, David H Lunt, Suzy CP Renn

**Affiliations:** 1 Department of Biology, Stanford University, Stanford, CA 94305, USA; 2 Department of Biology, Reed College, Portland, OR 97202, USA; 3 School of Biological Biomedical and Environmental Science, University of Hull, Hull HU6 7RX, UK; 4 Santa Catalina School, Monterey, CA 93940, USA

## Abstract

**Background:**

Gene duplication is a source of evolutionary innovation and can contribute to the divergence of lineages; however, the relative importance of this process remains to be determined. The explosive divergence of the African cichlid adaptive radiations provides both a model for studying the general role of gene duplication in the divergence of lineages and also an exciting foray into the identification of genomic features that underlie the dramatic phenotypic and ecological diversification in this particular lineage. We present the first genome-wide study of gene duplication in African cichlid fishes, identifying gene duplicates in three species belonging to the Lake Malawi adaptive radiation (*Metriaclima estherae*, *Protomelas similis*, *Rhamphochromis* “chilingali”) and one closely related species from a non-radiated riverine lineage (*Astatotilapia tweddlei*).

**Results:**

Using *Astatotilapia burtoni* as reference, microarray comparative genomic hybridization analysis of 5689 genes reveals 134 duplicated genes among the four cichlid species tested. Between 51 and 55 genes were identified as duplicated in each of the three species from the Lake Malawi radiation, representing a 38%–49% increase in number of duplicated genes relative to the non-radiated lineage (37 genes). Duplicated genes include several that are involved in immune response, ATP metabolism and detoxification.

**Conclusions:**

These results contribute to our understanding of the abundance and type of gene duplicates present in cichlid fish lineages. The duplicated genes identified in this study provide candidates for the analysis of functional relevance with regard to phenotype and divergence. Comparative sequence analysis of gene duplicates can address the role of positive selection and adaptive evolution by gene duplication, while further study across the phylogenetic range of cichlid radiations (and more generally in other adaptive radiations) will determine whether the patterns of gene duplication seen in this study consistently accompany rapid radiation.

## Background

Adaptive radiation, the evolution of genetic and ecological diversity leading to species proliferation in a lineage, is thought to be the result of divergent selection for resource specialization [1-3]. Differential selection in heterogeneous environments can result in adaptive radiation when there is a genetic basis for variability in organisms’ success in exploiting alternative resources [1-5]. Examples of such radiations include the Cambrian explosion of metazoans [6], the diversification of Darwin’s finches in the Galapagos [7], variations in amphipods and cottoid fishes in Lake Baikal [8], the Caribbean anoles [9], the Hawaiian Silverswords [10] and the explosive speciation of the cichlid fishes in the African Great Lakes [11].

The cichlid fishes are the product of an incredible series of adaptive radiations in response to the local physical, biological and social environment. While cichlids can be found on several continents [12], the most dramatic radiations are those of the haplochromine cichlids in the great lakes of East Africa. This speciose clade exhibits unprecedented diversity in morphological and behavioral characteristics [13] and accounts for ~10% of the world’s teleost fish. Interestingly, this clade also includes lineages that have remained in a riverine environment and have not radiated [14].

Classic work by Ohno [15] proposed a prominent role for gene duplication events in evolutionary expansion, despite their frequent loss due to drift [16]. Duplication makes extra gene copies available for dosage effects, subfunctionalization, or neofunctionaliztion [17], with the resultant phenotype potentially contributing to an organism’s fitness (for review see [18]). Current genomic research (e.g. primates: [19,20]) supports this, but the ability to compare closely related cichlid lineages that have and have not undergone an evolutionary radiation provides a critical tool for testing the association of gene duplication with adaptive radiation.

**Figure 1:**
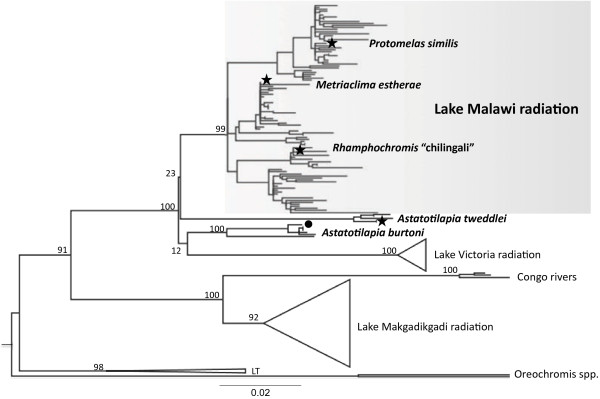
**Maximum likelihood phylogeny illustrating the positions of experimental (stars) and reference (circle) taxa.** The maximum likelihood tree is based on 1785 bp mitochondrial ND2. Nodes not supported by 50% maximum likelihood SH values collapsed and Lake Victoria, Lake Makgadikgadi, and Lake Tanganyika radiations are represented by triangles. The tree is rooted with *Oreochromis* and the scale bar indicates the mean number of nucleotide substitutions per site (DRYAD doi:10.5061/dryad.7vs2c).

We used array-based comparative genomic hybridization (aCGH) to identify gene duplications among 5689 genes for three Lake Malawi radiation species, which began accumulating molecular diversity approximately 5 million years ago [21] (*Metriaclima estherae*, *Protomelas similis*, *Rhamphochromis* “chilingali”) and one closely related riverine species from a non-radiated lineage (*Astatotilapia tweddlei*). While previous mitochondrial data suggested a bifurcation that separated the Lake Malawi radiation from the riverine species (Figure 1), more recent data based on ALFP data and single nucleotide polymorphisms derived from low coverage whole genome sequence [22-24] suggest that the Malawi flock is not monophyletic and that some of the riverine lineages may have contributed to Malawi genomes. These insights further support the use of *A. burtoni* as a reference to the three approximately equidistant test species. This is the first genome-wide study of gene duplication among haplochromine cichlids.

## Results

### aCGH identification of duplicated genes

**Figure 2:**
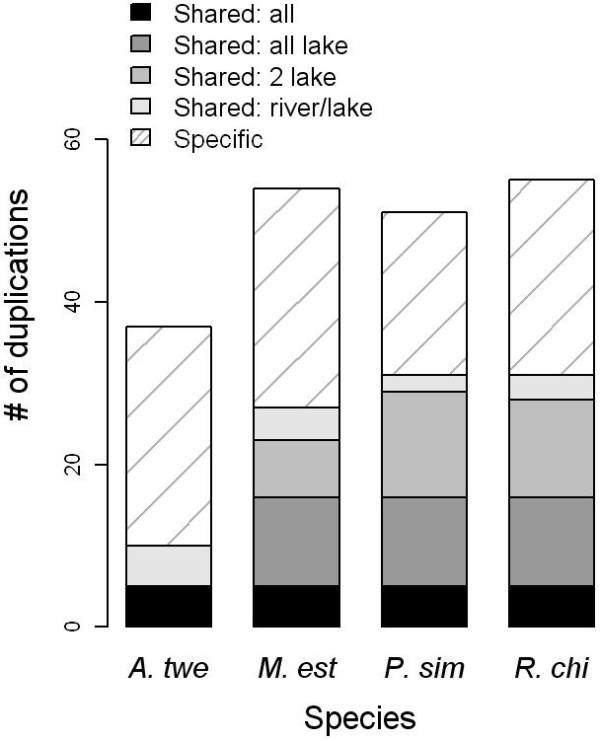
**Genes identified as duplicated among test species (P < 0.1 FDR). ***A. twe*: *A. tweddlei*; *M. est*: *M. estherae*; *P. sim*: *P. similis*; *R. chi*: *R.* “chilingali”. Shared: genes found duplicated in multiple species; Specific: genes found duplicated in only one species; lake: species belonging to the Lake Malawi radiation (*M. estherae*, *P. similis*, *R.* “chilingali”); river: the river species *A. tweddlei*.

Microarray features, representing a total of 5689 genes, passed quality control measures in all four test species. Among these, 145 array features (representing 134 genes) were determined to have an increased genomic content (*i.e.* copy number) for one or more heterologous species relative to *A. burtoni* (P < 0.1 FDR corrected) (Tables 1, 2). This included duplications of 54 genes in *M. estherae*, 51 in *P. similis*, and 55 in *R.* “chilingali”, compared to only 37 in *A. tweddlei*, the species from the non-radiated lineage (Figure 2). The number of duplicated genes identified for the species from the radiated lineage represents a 38%–49% increase relative to the number of duplicated genes identified in *A. tweddlei*. Consistent with their shared evolutionary history, shared duplications were prevalent among the three Lake Malawi species, with 11 duplications shared among all three and 16 duplications shared between two of the three species (Figure 2). Five genes had greater gene copy number in all four species relative to *A. burtoni*. Genes found duplicated in only one of the four species were also identified. This included 27 genes in *M. estherae*, 20 in *P. similis*, 24 in *R.* “chilingali” and 27 in *A. tweddlei*.

**Table 1:** **Genes duplicated relative to *A. burtoni *** **with informative BLAST hits**

**GenBank**	**Homology**	***A.twe***	***M.est***	***P.sim***	***R.chi***	**BitScore**
CN468828^2/2^	Adenine nucleotide translocator s598	ns	ns	ns	0.60	567
					0.0019	
DY630000	Alcohol dehydrogenase Class VI	ns	ns	0.73	ns	379
				0.0015		
DY630424	Alkylated DNA repair protein alkB homolog 7	ns	0.43	ns	ns	304
			0.0002			
DY629046	Arsenic (+3 oxidation state) methyltransferase	ns	ns	ns	1.06	150
					0.0031	
DY626788	ATPase, H + transporting, lysosomal V0 subunit E	ns	0.76	ns	ns	87.8
			0.0028			
DY628437	Claudin 29a (cldn29a) gene	ns	0.60	ns	ns	526
			0.005			
DY632040	Coiled-coil domain containing protein 80	ns	ns	1.19	2.13	434
				3E-05	5E-07	
DY629141	Crystallin gamma M2b	ns	ns	ns	0.43	829
					0.0024	
DY626204^1/2^	C-type lectin domain family 4 member C	ns	0.38	ns	ns	246
			0.0039			
DY631088	Cystatin-B	0.45	ns	ns	ns	150
		0.0016				
DY630353	Cytosolic sulfotransferase 3	ns	ns	0.62	0.64	713
				0.0015	0.0013	
CN470675	Dazl gene	ns	ns	ns	0.57	89.7
					0.0040	
DY629967^4/8^	Ferritin heavy subunit	ns	ns	ns	0.82	1160
					8E-05	
DY631817	Fish virus induced TRIM protein	ns	ns	0.590	ns	170
				0.0005		
DY626596	Fish virus induced TRIM protein	ns	ns	0.41	0.44	145
				0.0045	0.0030	
DY628624	Gamma M7 crystallin	ns	ns	ns	0.42	169
					0.0054	
DY630388	Glutamyl-tRNA(Gln) amidotransferase	0.48	ns	ns	ns	347
		0.0016				
DY626115^1/2^	GTPase IMAP family member 7	ns	ns	1.14	ns	370
				0.0016		
CN471284	High-mobility group 20B	0.60	ns	ns	ns	163
		0.0004				
CN469367	Hox gene cluster	1.34	1.16	0.86	1.11	183
		3E-05	9E-05	0.0006	0.0002	
DY627986	Hox gene cluster	1.81	1.12	0.80	1.22	95.1
		3E-07	1E-05	0.0001	5E-06	
DY629113	Immunoglobulin light chain	ns	ns	0.65	ns	482
				0.0025		
CN468953	Iron-sulfur cluster assembly enzyme ISCU	ns	ns	ns	0.86	610
					0.0022	
DY628151	Kallikrein-8 precursor	1.02	ns	ns	ns	102
		0.0015				
DY627800	Kinesin-like protein 2 (knsl2)	ns	0.86	1.84	1.14	398
			0.0013	9E-06	0.0003	
CN469578	KLR1 gene	1.04	ns	ns	ns	154
		4E-05				
DY629760	LOC100150543, polyprotein	1.35	ns	0.65	0.79	141
		8E-06		0.001	0.0003	
CN468718	LOC100151545, similar to Protein KIAA0284	0.72 0.0004	ns	ns	ns	145
DY629780	MHC class I	ns	0.84	1.26	1.05	161
			0.0053	0.0005	0.0016	
DY630620	MHC class IA antigen	ns	ns	0.42	ns	120
				0.0026		
DY630701	MHC class II alpha subunit	ns	ns	0.49	ns	764
				0.0053		
DY631898	MHC class II antigen alpha chain	ns	ns	0.94	ns	87.8
				0.0004		
DY631847	Mitotic spindle assembly checkpoint protein MAD2A	0.60	ns	ns	ns	374
		0.0008				
DY627079	Muscle-type creatine kinase CKM2	ns	0.41	ns	ns	787
			0.0049			
CN469375^2/3^	Peptidyl-prolyl cis-trans isomerase NIMA-interacting 1	ns	0.69	ns	ns	663
			0.0003			
DY628779	Post-GPI attachment to proteins factor 2	ns	0.87	ns	ns	123
			0.0002			
DY626114^2/2^	Ras association domain-containing protein 4	ns	0.82	ns	ns	1086
			0.0001			
DY630104	Ras-related C3 botulinum toxin substrate 2	1.44	0.83	1.47	1.90	331
		8E-06	0.0003	7E-06	1E-06	
DY630508^1/3^	Replication factor C subunit 5	1.04	ns	ns	ns	1234
		2E-05				
DY628495	Ribosomal protein, large P2 (60S)	ns	ns	ns	1.01	161
					0.0001	
DY630832^2/3^	Ribosomal protein S20 (40S)	ns	0.65	ns	ns	663
			0.001			
DY626643	Serine/threonine phosphatase gene	ns	0.57	0.57	0.54	87.8
			0.0004	0.0004	0.0063	
CN470072	Sestrin 3	ns	1.30	1.61	1.70	116
			0.0007	0.0002	0.0002	
DY629126	Short coiled-coil protein	ns	ns	ns	0.59	242
					0.0025	
DY630540	Small inducible cytokine SCYA102	ns	0.64	ns	ns	1204
			0.0019			
CN471492	Solute carrier family 9 (sodium/hydrogen exchanger)	ns	ns	0.63	ns	197
				0.0021		
CN471103^1/3^	Ubiquitin	ns	ns	1.27	ns	985
				0.0042		
DY629776	UDP glycosyltransferase 2 family, polypeptide A1	ns	0.92	ns	ns	304
			0.0006			
CN469822	Vacuolar ATP synthase subunit G 1	0.79	ns	ns	ns	277
		0.0007				
DY632057	Pituitary adenylate cyclase activating polypeptide receptor 1A	ns	1.73	1.98	1.79	170
DY626009	Non-LTR retrotransposon Rex1a	0.70	ns	ns	ns	82.4
		0.002				
DY629391	Non-LTR retrotransposon Rex3_Tet	0.94	ns	ns	ns	122
		0.0028				
DY631649	SINE sequence	ns	0.78	ns	ns	138
			0.0002			

Genomic Content Value: Log2 Hybridization coefficients for each species relative to *A. burtoni* as estimated by the linear model following background correction and normalization followed by the uncorrected p-value for those significantly different (P < FDR 0.1). BitScore: the quality of the alignment for the annotated homology. *A.twe*: *A. tweddlei*; *M.est*: *M. estherae*; *P.sim*: *P. similis*; *R.chi*: *R.* “chilingali”; “ns”: not significant; superscript numbers: when multiple array features represent a gene, these numbers indicate the number of significant array features and the total number of array features for that gene represented in the table by a single GenBank number and data row.

**Table 2:** **Genes duplicated relative to *A. burtoni *** **with no informative BLAST hit**

**GenBank**	***A.twe***	***M.est***	***P.sim***	***R.chi***
CN469125^2/2^	1.32	ns	ns	ns
	2E-5			
CN469431	ns	ns	ns	0.50
				0.0017
CN469460	ns	ns	0.64	ns
			0.0007	
CN469913	ns	ns	ns	0.63
				0.0008
CN470216	ns	0.39	ns	ns
		0.0039		
CN470402	ns	ns	0.48	0.45
			0.0034	0.0051
CN470540	ns	ns	ns	0.60
				0.0051
CN470597	0.65	ns	ns	ns
	0.0013			
CN470646	0.73	ns	ns	ns
	0.0003			
CN470701	ns	ns	ns	0.65
				0.0052
CN470713	ns	ns	0.68	ns
			0.0009	
CN470724^1/2^	1.02	ns	ns	ns
	0.0027			
CN470781	0.58	ns	ns	ns
	0.0025			
CN470835	ns	1.55	ns	ns
		0.0002		
CN470857	ns	0.67	ns	0.96
		0.005		0.0006
CN470988	ns	1.28	ns	1.34
		0.0007		0.0005
CN471261	ns	ns	0.93	ns
			0.001	
CN471811	ns	1.35	1.22	ns
		6E-6	1E-5	
CN471851	ns	ns	0.47	ns
			0.0018	
CN472050	ns	ns	ns	0.70
				0.0002
DY625804	ns	1.23	ns	ns
		0.0001		
DY625845^1/2^	1.16	ns	ns	ns
	6E-6			
DY625884	0.49	ns	ns	ns
	0.0024			
DY625919	1.18	ns	ns	ns
	0.0001			
DY626122	1.50	ns	ns	ns
	2E-5			
DY626140	ns	1.05	ns	ns
		0.0036		
DY626192	ns	0.75	ns	ns
		0.0002		
DY626304	ns	ns	0.99	1.50
			4E-5	2.2E-6
DY626389	ns	ns	0.57	ns
			0.0022	
DY626428	ns	ns	ns	0.39
				0.0053
DY626737	ns	ns	0.79	ns
			0.0001	
DY626766	ns	0.81	0.98	0.68
		0.0003	0.0001	0.0009
DY627085	ns	0.44	ns	ns
		0.0043		
DY627087	1.15	ns	ns	ns
	3E-5			
DY627338	0.72	ns	ns	ns
	0.0006			
DY627361	ns	ns	ns	0.74
				0.0001
DY627641	ns	0.76	0.85	ns
		0.0012	0.0006	
DY627780	ns	1.51	ns	ns
		3E-6		
DY627911	1.04	0.87	0.49	ns
	5E-5	0.0002	0.0046	
DY628148	0.50	ns	ns	ns
	0.0012			
DY628172	1.38	ns	ns	ns
	4E-6			
DY628268	ns	ns	0.46	ns
			0.0017	
DY628316	ns	0.64	ns	ns
		0.0026		
DY628477	ns	ns	ns	0.58
				0.0052
DY628517	0.76	ns	ns	ns
	0.0002			
DY628561	ns	ns	ns	0.42
				0.0052
DY628642	ns	ns	1.62	1.13
			9E-5	0.0087
DY628702^1/2^	ns	ns	ns	0.67
				0.0004
DY628714	ns	ns	ns	0.48
				0.0027
DY629058	ns	ns	ns	2.41
				3.7E-5
DY629123	ns	0.87	0.83	1.41
		0.0001	0.0001	3.8E-6
DY629387	ns	ns	1.18	ns
			4E-5	
DY629482	1.39	0.71	ns	1.12
	1E-5	0.0008		4.3E-5
DY629717	ns	ns	1.60	1.28
			0.0002	0.0007
DY629882^1/2^	ns	ns	ns	0.77
				0.0032
DY629912	1.41	2.21	1.06	1.16
	8E-6	0.0003	7E-6	1.1E-6
DY630229	ns	0.88	0.83	ns
		0.0015	0.0021	
DY630284	ns	0.54	ns	ns
		0.0032		
DY630373^1/2^	ns	0.89	1.00	1.23
		0.0001	6E-5	1.5E-5
DY630867	0.97	0.64	ns	ns
	0.0002	0.0024		
DY630964	ns	ns	ns	0.95
				0.0037
DY630993	ns	0.67	ns	ns
		0.002		
DY631067	ns	0.78	0.80	1.02
		0.0008	0.0007	0.0002
DY631315	ns	ns	1.57	1.16
			0.003	0.0019
DY631408	ns	ns	0.50	ns
			0.0033	
DY631442	ns	1.40	1.16	ns
		0.0014	0.004	
DY631505^1/2^	ns	0.72	ns	ns
		0.0029		
DY631507	0.67	0.69	0.78	0.61
	0.0013	0.0011	0.0005	0.0022
DY631680	ns	ns	ns	1.02
				0.0019
DY631698	ns	ns	1.03	ns
			0.0006	
DY631821	ns	ns	0.61	0.60
			0.0038	0.0039
DY631827	ns	0.86	ns	ns
		0.0046		
DY631850	0.85	ns	ns	ns
	0.0009			
DY631869	ns	0.39	ns	ns
		0.0038		
DY632007	0.99	ns	ns	ns
	0.0002			
DY632058^3/3^	ns	0.90	0.72	0.71
		0.0007	0.0026	0.0028
DY632092	ns	1.09	ns	ns
		0.0007		
DY632097	ns	0.79	ns	0.82
		0.0031		0.0002
DY632134^2/2^	0.94	0.86	ns	0.84
	0.0002	0.0003		0.0003
DY632256	ns	ns	1.56	ns
			0.0033	
DY632294	ns	0.41	ns	ns
		0.0035		

Genomic Content Value: Log2 Hybridization coefficients for each species relative to *A. burtoni* as estimated by the linear model following background correction and normalization followed by the uncorrected p-value for those significantly different. *A.twe*: *A. tweddlei*; *M.est*: *M. estherae*; *P.sim*: *P. similis*; *R.chi*: *R.* chilingali; “ns”: not significant; “*”: the GenBank number is a representative for multiple array features for that gene.

In twenty cases, the gene identified as duplicated was represented on the array by multiple features. Five of these instances showed complete concordance among the two or three array features representing that gene such that all showed the same significant pattern across species. However, for some genes found to be duplicated, only one of the two (n = 8), one of the three (n = 4), two of three (n = 2) or in one case four of the eight array features representing that gene reached statistical significance. In most cases, those features that did not reach statistical significance followed a similar pattern (Additional file 1 Figure S1). However this was not always the case which may be due to different parts of the gene sequence being represented by the different features, high variance or poor quality for one of the features, miss-annotation of the array, or other technical reasons.

BLAST comparison of array feature sequence similarity to the nucleotide database allows annotation and predicted function for discussion of possible adaptive processes. Based on these annotations, several candidate genes were identified as duplicated in and among lineages. Repeated similarities of functional annotations were noticed, particularly for genes involved in immune response, ATP metabolism and detoxification.

### Quantitative PCR verification

**Figure 3:**
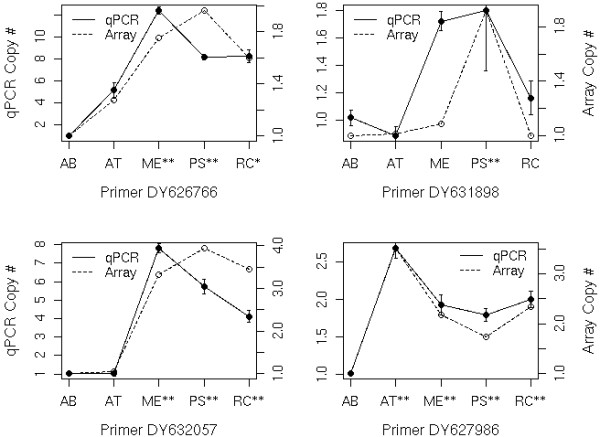
**qPCR validates gene copy number determined by aCGH.** Abbreviations are genus and species initials. Primer loci are named for the Genbank number of the *A. burtoni* array feature sequence. **P <0.1 FDR, *P <0.2 FDR found by array analysis.

Four loci found to be duplicated in one or more test species according to aCGH were chosen for quantitative PCR (qPCR) validation for their observed duplication patterns- one duplicated in all species relative to *A. burtoni*, two duplicated in all three Lake Malawi radiation species and one species-specific duplication (Table 2). Primer pairs that were designed to *A. burtoni* sequence successfully amplified product with a similar or slightly reduced efficiency in each heterologous species tested (Table 2). We estimated the copy number relative to *A. burtoni* for these loci based on the array hybridization ratio, and compared that to the copy number estimated from the qPCR results. Each duplication of a given locus as identified by the microarray analysis also showed significantly increased copy number of that locus according to the qPCR analysis (Figure 3). Furthermore, the pattern of relative copy number among test species observed in the qPCR analysis, reflected, with few exceptions, the pattern of relative copy number observed in the microarray analysis. The only notable discrepancy was an increased genomic content for gene DY631898 detected for *M. estherae* that was not found by microarray analysis.

## Discussion

Gene duplication is an important source of functional novelty and has a demonstrated role in adaptive evolution [18]. Such adaptations can allow for niche diversification, as has been suggested for thermal adaptation (plants: [25], Antarctic ice fish: [26]) and for metabolic novelty (C–4 photosynthesis: [27]). The adaptive radiations of the African cichlid fishes exhibit remarkable niche exploitation in the presence of low levels of sequence divergence (reviewed by [13,21]). However, little is known regarding the relative number of duplicated genes, nor the identity of duplicated genes, within this group. If there is an increased rate of gene duplication or gene duplicate retention in radiated lineages, or if particular duplications are associated with these lineages, then their pattern and identity could provide insight into the processes facilitating the rapid expansion of the African cichlids. The patterns reported and validated here indicate shared and increased gene duplication within the Lake Malawi radiation compared to a close non-radiating lineage. While three of the identified gene duplicates were annotated as mobile elements (retrotransposons or SINE element), the majority of the genes could be assigned functional annotation based on a manually curated homology search to UniProtKB/Swiss-Prot for those genes found to be duplicated. Based on individual gene names and functional annotations, several candidate genes, including those that are involved in immune response, ATP metabolism and detoxification, are identified as duplicated in and among lineages (Table 1). Some of these gene duplicates may underlie adaptive phenotypic change.

### Immune response

The evolution of immune response is a potent factor contributing to the divergence of lineages, resulting from strong selection on certain loci [28-30]. A greater number of genes associated with immune response (4–9) are found to be duplicated in the Lake Malawi lineage as compared to the riverine species (2). This list includes two finTRIM genes (one duplicated in *P. similis* and the other in both *P. similis* and *R.* “chilingali”), a gene family that is known to play a role in immunity against viral infection, and several finTRIM paralogs have been found in teleost fishes, resulting from duplication and positive selection (70 in trout, 84 in zebrafish) [31]. There are also five major histocompatibility complex (MHC) genes- two MHC class I, two MHC class II, and kinesin-like protein 2- found duplicated in one or more of the species from the radiated Lake Malawi lineage. The MHC gene family, in addition to being involved in immunity (salmon: [32]), has a history of expansion and contraction through duplication and deletion [33]. MHC gene families vary in size among teleosts, with particularly large families in cichlids [34-38]. Additional immune related genes duplicated in the Lake Malawi radiation include an immunoglobulin light chain, small inducible cytokine (associated with the MHC region in stickleback: [39]), and sestrin 3. In *A. tweddlei*, the test species from the non-radiated lineage, two immune genes, kallikrein-8 and natural killer cell lecin-type receptor, are also found to be duplicated. The identification of several duplicated immune function genes is consistent with previous work documenting size variability and rapid expansion of immune function gene families (Drosophila: [28], silkworm: [40]) that may allow species to invade new niches or better adapt to existing ones.

### ATP metabolism

ATP metabolism and function is critical to many physiological processes. Two ATP synthases and one ATP transporter are found duplicated among the four species. Subunits G and E of vacuolar ATPases, which couple the energy of ATP hydrolysis to proton transport across intracellular and plasma membranes, are duplicated in *A. tweddlei* and *M. estherae*, respectively. In *R.* “chilingali”, the adenine nucleotide translocator (ANT) s598 is found duplicated. This mitochondrial transmembrane protein is the most abundant mitochondrial protein and is integral in the exchange of ADP and ATP between the mitochondria and the cytoplasm. Increased expression of mitochondrial ATP synthase has been found in cold acclimated carp [41] and ANT genes are being studied for their potential adaptive role in thermal acclimation (fugu: [42]). Given that these ATP synthase and transport genes are found duplicated in all 4 species of this study rather than showing enrichment only within the Lake species, they may represent an ancestral duplication, or deletion in *A. burtoni*, nonetheless, their retention may be associated with adaptation to ecological conditions.

### Detoxification

Selection on duplicated detoxification genes (those involved in the breakdown of toxic compounds) can determine survival in particular environments or can contribute to expansion into new niches. One example is seen in plant-herbivore interactions, where gene duplication has been implicated in the ability of herbivores to detoxify plant defense compounds and prevent exclusion of the herbivore from that food source [43,44]. We detect duplication of detoxification genes in all three species from the radiated lineage. In *P. similis* and *R.* “chilingali”, the sulfotransferase (SULT) gene cytosolic sulfotransferase 3 is found duplicated. SULT genes are detoxifying enzymes that catalyze the transfer sulfonate groups to endogenous compounds and xenobiotics. Once sulfated, compounds may become more easily excreted from the body. In zebrafish, ten SULT proteins have been cloned, two of which show strong activity towards environmental estrogens [45]. Zebrafish SULTs have also been found to act on other xenobiotics [46]. In Atlantic cod, a SULT gene was found to be upregulated in response to polluted water [47]. In *R.* “chilingali”, two other genes involved in detoxification, arsenic methyltransferase and ferritin (heavy subunit), are found duplicated. Arsenic methyltransferase converts inorganic arsenic into less harmful methylated species, and ferritin is an iron storage protein that is essential for iron homeostasis, keeping iron concentrations at non-toxic levels. Another iron-related protein, the iron-sulfur cluster assembly enzyme, was also duplicated in *R.* “chilingali”. It is possible that some of these gene duplicates have been retained due to a selective advantage for metabolic breakdown of environmental compounds and toxins. Such duplicates may allow novel physiological interactions with the chemical, physical and pathogenic environment that may play a role in adaptive divergence as a lineage radiates to inhabit new niches such as those associated with the African Great Lakes.

### Gene family membership

Gene families by their very nature reveal a propensity for duplication and duplicate retention of certain genes. One study estimated that 38% of known human genes can be assigned to gene families, based on amino acid sequence similarity [48]. These gene families typically consist of two genes, but the largest gene families can have more than 100 members. In the present study, several of the genes found to be duplicated were members of large gene families, comprised of multiple known genes. These include 40 S and 60 S ribosomal proteins (duplicated in *R.* “chilingali” and *M. estherae*), claudin 29a (*M. estherae*), GTPase IMAP family member 7 (*P. similis*), C–type lectin domain family 4 (*M. estherae*), high-mobility group 20B (HMG20B) from HMG-box superfamily (*A. tweddlei*), and hox gene cluster genes (all species). Hox genes are important in the regulation of development, and have been found to be associated with differential jaw development in cichlid fishes [49,50]. An immunoglobulin light chain gene belonging to the largest gene family represented in this study was found duplicated in *P. similis*. Since large gene families are comprised of multiple paralogs and may possess a greater tendency for expansion, it is not surprising that large gene families are well represented in our list of duplicated regions.

### qPCR verification

The robust validation of aCGH results using quantitative PCR not only verifies the increased genomic content for all four loci analyzed in test species relative to *A. burtoni,* it also provides a complementary approach that may prove to be a more efficient means to survey candidate loci in future population level analyses. For each locus except DY631898, the pattern of copy number among the four test species relative to *A. burtoni* is similar to that found by aCGH. However, the copy number estimated by qPCR differs from that estimated with array results. This is particularly true of the DY626766 and DY632057 loci, which showed greater qPCR copy number than predicted, despite the underestimation bias possible for those loci. Similarly, in *M. estherae*, the DY631898 locus appeared to be substantially higher in copy number than predicted by the array results. This discrepancy could result from three factors. First, it may be due to the fact that aCGH will produce an underestimate of true copy number when there is sequence divergence of the heterologous species relative to the platform provided the primers are in a conserved sequence region. Second, while qPCR and microarray analyses both provide relative rather than absolute measures, the scale of the relationship measurements may differ due to the difference in normalization techniques applied to the raw data. Finally, particularly for the case of the DY631898 locus in *M. estherae*, the micorray analysis includes only two replicates for each species and is thus sensitive to technical error where technical failure of qPCR is more easily replicated. Nonetheless, even for the two instances in which reduced primer efficiency in the tested heterologous species would have been expected to result in an underestimate rather than an overestimate of copy number, the pattern identified by aCGH was upheld. Regardless of discrepancies in magnitude, our quantitative PCR results demonstrate, with the exception of one data point, both qPCR,and aCGH are valid techniques for estimation of relative copy number in heterologous species. While aCGH allows one to survey a greater number of genes, the qPCR technique may provide an efficient means to assess copy number variation (CNV) of candidate loci within a larger population in order to illuminate the role of gene duplication on a microevolutionary scale.

### Technical considerations

The use of aCGH was initially developed for cancer studies and has been applied to several within species studies, but has less frequently been used to assess between species patterns of gene duplication. Careful consideration of the technical biases and conservative interpretation of the results are warranted [51,52]. The array features analyzed represent only 5689 genes, a fraction (25-30% of a standard vertebrate genome) of predicted total gene content for these species. Furthermore, because genomic content for each gene has been assessed relative to the array platform species *A. burtoni*, any gene that has equivalent copy number (even if greater than 1) in both the platform and the heterologous species will go undetected. Similarly, those genes that appear to be duplicated in all heterologous species may actually represent a reduction in genomic content in *A. burtoni* due to gene deletion events. Furthermore, aCGH with spotted cDNA arrays does not allow quantification among different genes and it is therefore impossible to provide absolute copy numbers. We identify five such genes, two annotated as Hox gene cluster genes, one as a Ras-related C3 botulinum toxin substrate gene and two that lack annotation, that appear to be duplicated in all four test species, but which may in fact be deleted in *A. burtoni*. In our study we do not attempt to distinguish between these two scenarios.

The hybridization bias due to sequence divergence of the heterologous species from the platform species is another an important consideration for the interpretation of aCGH results. Diverged sequences will hybridize less well to the array feature than *A. burtoni* DNA. Therefore, it follows that duplicated genes for which the paralog is highly diverged will be less likely to be detected as duplicated than duplicated genes with paralogs that are less diverged from the platform species, as found by Machado and Renn [52]. Therefore, older gene duplication events, those with very little purifying selection pressure, and those with strong positive selection in the gene region represented on the array are less likely to be identified, while recent duplication events or highly conserved duplicates are more likely to be identified. Therefore, the results presented here represent a subset of the total gene duplicates that may differ from the subset of gene duplicates identified by other techniques such as sequence assembly or depth of coverage. Gene number and gene copy number identified by short read sequencing technology is prone to overestimation of copy number variation [53]. Nonetheless, the numbers reported here are clearly an under-representation of the total and may present a different phylogenetic pattern of retention than other subsets of gene duplicates.

In this study, we use a recent adaptive radiation so that, whilst strong positive selection on duplicates might be overlooked by the aCGH technique, the majority of very recent duplications are likely to be identified. We find a pattern of increased gene duplication in these Lake Malawi haplochromines, with 38-49% more genes duplicated than in the non-radiated lineage. Care must be taken in interpreting this increase in the context of adaptive radiation, with four primary considerations. First, only a subset of genes (*i.e.* those present on the array with available sequence) was tested. Second, gene duplicates may have become fixed in ancestral populations due to neutral processes such as founder events, genetic bottlenecks or drift during the relatively recent evolutionary past. Sequence data from multiple species will be necessary to distinguish neutral vs. adaptive evolutionary processes. Third, due to the shared evolutionary history of the three Lake Malawi species, they cannot be considered independent. Fourth, the ecology of the species, lake versus riverine, is confounded with the tendency to radiate. Therefore, as tantalizing as these results are, our single comparison of radiated versus non-radiated lineages requires further support before general patterns associated with adaptive radiation can be rigorously discussed. Fortunately, the African cichlids provide such a system with which to undertake this [14].

## Conclusions

Only recently have studies begun to examine the patterns of gene duplication and copy number polymorphism across species in natural systems, beyond primates (e.g. [26,54-56]). While other studies have examined specific genes (e.g. [57-59]), we present the largest analysis thus far of genome wide patterns of gene duplication across lineages of the African cichlid radiations. We identify several candidate gene duplicates in four cichlid species and find a pattern of increased gene duplication within the Lake Malawi radiation. While our inference regarding the adaptive value of candidate gene duplicates must be tempered, the results of this study support the hypothesis that gene duplication, particularly of genes related to immune response, ATP metabolism and detoxification, is a characteristic of the Lake Malawi adaptive radiation. Assessment across a greater phylogenetic range of cichlid radiations will identify consistent patterns of gene duplication correlated with radiated and non-radiated lineages, and comparative sequence analysis will reveal the potential contribution of natural selection to gene duplicate evolution.

## Methods

### aCGH identification of duplicated genes

Genomic DNA, extracted from ethanol-preserved field tissue samples (n = 2 per species) by standard ProteinaseK/Phenol protocol, was size reduced by Hydroshear (Genome Solutions/Digilab) to 1–5 Kb. DNA (4 μg) and labeled with Alexa-Fluors (555 & 647) conjugated dCTP by Klenow polymerization (Invitrogen, BioPrime® Direct Array CGH Genomic Labeling System catalog# 18095–011). Each species was hybridized twice (once with each individual) (in dye swap) against a reference pool of *A. burtoni* genomic DNA using the *A. burtoni* cDNA PCR product spotted microarray which contains ~20,000 features, representing ~16,000 unique sequences of which ~65% have available EST sequence [60] (GEO platform GPL6416). After a 16 hour hybridization (67.5°C, 3.4× SSC, 0.15% SDS, 1 mM DTT, Cot-1DNA), arrays were washed and scanned (Axon 4100B, Genepix).

Microarray data (GEO series GSE19368) were filtered by omitting features with a lack of sequence information, known ribosomal content, or that had faint array signal (<2 SD above background). Only features that survived this quality control for all eight microarrays were analyzed. Data were corrected for background intensity (“minimum”) and were loess normalized within array using 250 conserved features [60]. This corrects for bias introduced by sequence divergence under standard normalization [61]. Duplicated genes were identified as those with increased fluorescence according to the “lmFit” statistical model with “eBayes” correction and FDR adjustment for P < 0.1 significance level [62]. The reported results are underestimates of duplication levels, due to the fact that diverged duplicates are less likely to be detected [52]. GEL_50_ measurements [63] indicated that experiments were of similar statistical power (*M. estherae*: 1.80, *P. similis*: 1.95, *R.* “chilingali”: 1.61, *A. tweddlei*: 1.89). The automated annotations available from DFCI were not used in this study because many proved to be uninformative. Instead, functional annotations for genes were gathered only for identified duplicates using BLASTn to compare EST sequences to the UniProtKB/Swiss-Prot database. The top 100 hits were returned in order to identify informative annotations and infer function based on homology. Bit scores are reported for these annotations. No filtering or masking was applied during the BLASTn thus annotations for repetitive sequences and transponsons are included.

### Quantitative PCR

Genomic content was validated for four genes using qPCR (Table 3). gDNA concentration was quantified with 1.5× SYBR Green I (Roche Applied Science) on a Nanodrop 3300 (Thermosavant). Triplicate qPCR reactions (Opticon MJ Research) contained 0.75× SybrGreen, 1× Immomix (Biolabs), 200–500 nM primers and 0.2 ng sample DNA in 10 μl reactions (95°C– 10 min; 35 cycles of: 94°C– 2 min, 60°C- 20 sec, 72°C- 15 sec, and 2 min extension). Copy number relative to *A. burtoni* was calculated as CT, the cycle number at a set threshold relative to the *A. burtoni* standard curve, standardized to an *A. burtoni* copy number of 1. Primer efficiency was calculated with a dilution series for *A. burtoni* DNA and one test species (Additional file 2: Table S2).

**Table 3:** Oligonucleotide primers used for qPCR designed against GenBank sequence available for microarray features

**GenBank**	**Primer sequence**	**Homology**		**Primer efficiency**	
			**Predicted length**	***A. burtoni***	**Test species**
DY626766	F: TCGGTCTCCTTAACCGGATG	No Hit	193	86	74
	R: CTGAGTTTGGCTGCCCGTAA				*(P. similis)*
DY627986	F: ACGAACACCCGAACGGAAAC	Hox gene cluster	222	100	104
	R: GGTGCACGCACATGAACTGT				*(M. estherae)*
DY631898	F: CGTCCCAGTGAGGATGAGGA	MHC class II antigen	161	82	82
	R: TGATGCTGATCGGTTGATGC				*(R.* “chilingali”*)*
DY632057	F: ATTACTGCGAGTGCCGTCCA	Pituitary adenylate cyclase activating	150	91	78
	R: CTGCGCCCTGAAAGAACAGA	polypeptide receptor 1A			*(A. tweddlei)*

Primer Efficiency: percent is based on 4-fold template dilutions for *A. burtoni* and one heterologous test species indicated in parentheses.

### Availability of supporting data

The data sets supporting the results of this article are available in the GEO repository, [GSE19368: http://www.ncbi.nlm.nih.gov/geo/query/acc.cgi?acc=GSE19368) and DRYAD (doi:10.5061/dryad.7vs2c http://datadryad.org/resource/doi:10.5061/dryad.7vs2c).

## Competing interests

The authors declare that they have no competing interests.

## Authors’ contributions

SCPR, DHL, DJ conceived of the project. HEM, CRLR, GJ performed the experiments. HEM conducted the analyses. SCPR, HEM, DHL prepared the manuscript. All authors read and approved the final manuscript.

## Supplementary Material

Additional file 1: Figure S1 Genes Identified as duplicated that are represented on the array by more than one microarray feature. Those with perfect concordance for significance calls (n = 5) are not shown. In each plot, the y-axis represents Log2 hybridization ration for the heterologous species relative to *A. burtoni*. Each line indicates an individual microarray feature that is statistically significant (P < FDR 0.1, black), marginally significant (P < FDR 0.2, solid grey) or not significant (grey dashed) for genes represented by 2 microarray features (A-H), 3 microarray features (I-N) and 8 microarray features (O). AT: *A. tweddlei*; ME: *M. estherae*; PS: *P. similis*; RC: *R.* chilingali. Click here for file

Additional file 2: Table S2 Oligonucleotide primers and efficiency used for qPCR designed against GenBank sequence available for microarray features. Click here for file
