# Supplementary figures and images for: Gene duplication in an African cichlid adaptive radiation
Source: BMC Genomics. 2014 Feb 26;15:161. doi: 10.1186/1471-2164-15-161 (PMC3944005; doi:10.1186/1471-2164-15-161)

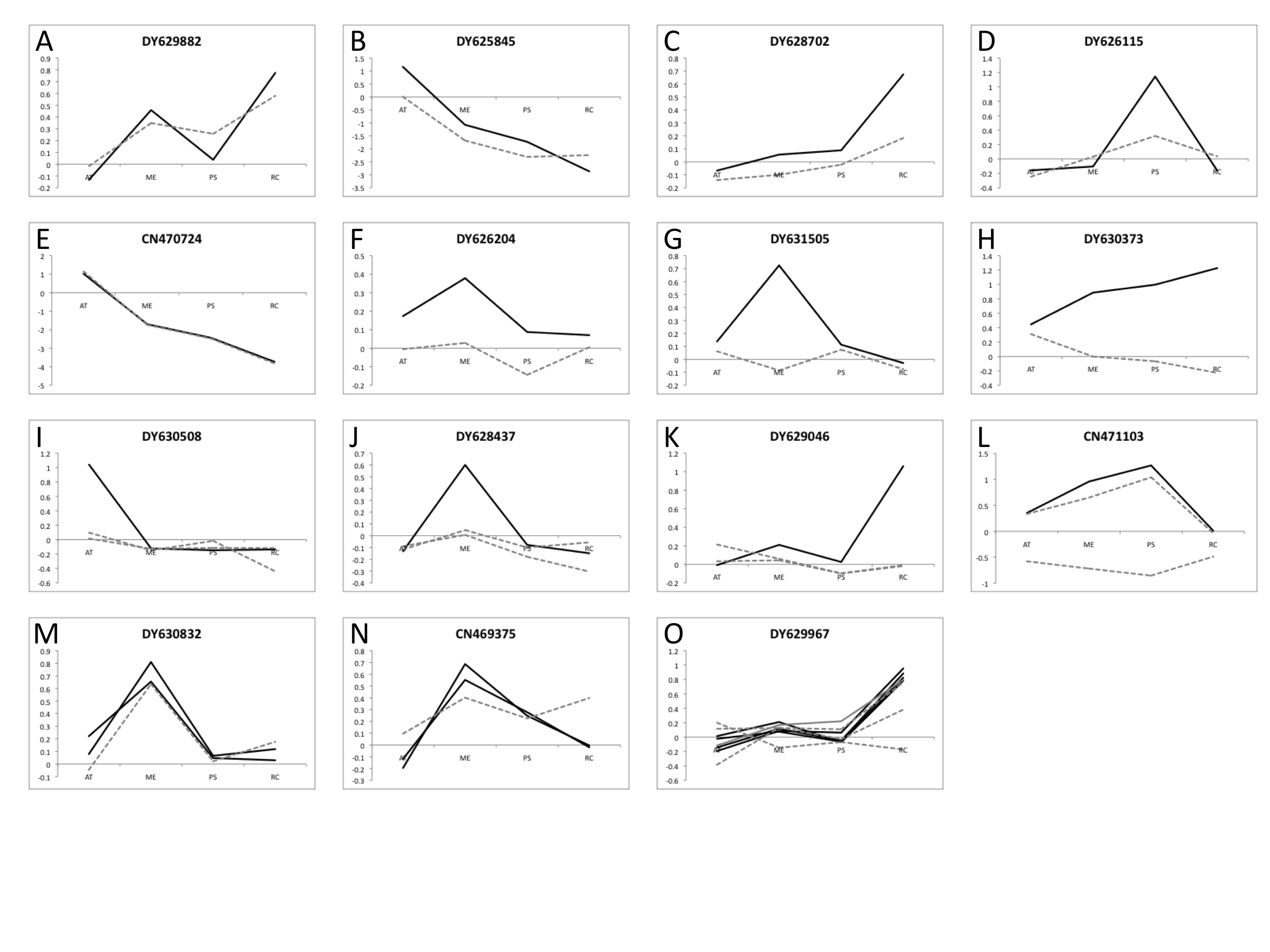

Supplement: Additional file 1: Figure S1 — Genes Identified as duplicated that are represented on the array by more than one microarray feature. Those with perfect concordance for significance calls (n = 5) are not shown. In each plot, the y-axis represents Log2 hybridization ration for the heterologous species relative to A. burtoni. Each line indicates an individual microarray feature that is statistically significant (P < FDR 0.1, black), marginally significant (P < FDR 0.2, solid grey) or not significant (grey dashed) for genes represented by 2 microarray features (A-H), 3 microarray features (I-N) and 8 microarray features (O). AT: A. tweddlei; ME: M. estherae; PS: P. similis; RC: R. chilingali. [file 1471-2164-15-161-S1.jpeg]
